# Residual Dentin Thickness of Bifurcated Maxillary Premolars Following Two Post Space Preparation Methods

**Published:** 2013-08-01

**Authors:** Jamileh Ghoddusi, Ali Bagherpour, Fatemeh Mahmudabadi, Maryam Forghani, Majid Sarmad

**Affiliations:** a Department of Endodontics, Dental Research Center, Dental School, Mashhad University of Medical Sciences, Mashad, Iran; b Department of Oral and Maxillofacial Radiology, Dental School, Mashhad University of Medical Sciences, Mashhad, Iran; c Private Practice, Mashhad, Iran; d Department of Endodontics, Dental Material Research Center, Dental School, Mashhad University of Medical Sciences, Mashhad, Iran; e Department of Statistics, Mathematical Sciences School, Ferdowsi University of Mashhad, Mashhad, Iran

**Keywords:** Bifurcated Maxillary Premolars, Cone-Beam Computed Tomography, Residual Dentin Thickness, Root Canal Therapy

## Abstract

**Introduction:**

The aim of this in vitro study was to compare the effect of Gates-Glidden and Peeso reamer drills on residual dentin thickness during post space preparation in order to discover which method has minimum root structure damage.

**Materials and Methods:**

Thirty extracted human maxillary premolars with bifurcations at root middle were horizontally cut 15 mm coronal to the apical end after root canal treatment. The samples were scannedby Cone Beam Computed Tomography (CBCT) before and after preparing the post space. Residual dentin thicknesses were measured at 4-, 6-, and 8-mm levels from the apex. Data were analyzed using repeated measured ANOVA.

**Results:**

Endodontic therapy and post space preparations removed more dentin within the bifurcation of both roots compared to outer dentin. The difference in residual dentin thickness was highly significant regarding stage (before and after post space preparation) in all levels and stage × device in coronal and middle levels (P<.05). This in vitro study emphasizes the minimal dentin width in the buccal root of maxillary premolars, especially near the bifurcation.

**Conclusion:**

Lack of adequate residual dentin thickness after post space preparation implies that the use of posts in maxillary first premolars should be limited. When mandatory, it is recommended that post space be prepared with Gates-Glidden drill in the palatal root of maxillary first premolars and use of Peeso reamer be avoided.

## Introduction

Vertical root fractures (VRFs) occur mostly in endodontically treated and restored teeth [1]. Some factors such as excessive removal of dentin during root canal instrumentation [2, 3] and post space preparation [4] have been identified as causes of VRF. A drill with a non-cutting end such as Gates-Glidden or Peeso reamer, which will preferentially remove the softer gutta-percha rather than the canal wall dentin, should be used for post space preparation [5].

Location and direction of VRF are influenced by residual dentin thickness (RDT), external root morphology and canal curvature [6]. With regard to external root morphology, the roots with narrow mesiodistal diameter compared to the buccolingual dimension (such as maxillary and mandibular premolars) are most susceptible to fracture [7, 8].

The proximal root depression, the reduced wall thickness, and the decreased radius of curvature in the palatal aspect of the buccal root of maxillary premolars increase fracture susceptibility in these roots [9]. Post space preparation, especially in VRF-susceptible teeth, is very important because of their specific cross-section contours and curvatures [7, 10]. The residual dentin thickness after post space preparation should be a minimum of 1 mm around the entire circumference of the canal [11, 12]. Katz *et al.* reported that post space preparation with ParaPost drills in the bifurcated maxillary premolars resulted in RDT values less than one millimeter in the palatal aspect of the buccal roots [13].

The aim of this *in vitro *study was to evaluate and determine the residual dentin thickness in the bifurcated roots of maxillary premolars after post space preparation with Gates-Glidden burs or Peeso reamer drills.

## Material and Methods

All procedures in this study were carried out according to protocols approved by the Ethics Committee of Mashhad University of Medical Sciences, Mashhad, Iran. Thirty human bifurcated maxillary first premolars were collected from patients were orthodontically extracted. The inclusion criteria were closed apices, free from caries, restorations and canal obstruction. In all the teeth, the bifurcation was not located more apically than the middle third of the root. After extraction, the teeth were immersed in 5.25% NaOCl for 30 min in order to disinfect them. Two x-ray images were obtained from the buccal and mesial surfaces to record furcation anatomy.

### Root Canal Preparation

Root canal preparation was performed by one practitioner. Access cavities were prepared with a diamond bur. The working length was determined by insertion of a #10 K-file (Dentsply Maillefer, Ballaigues, Switzerland) into the canal until the file tip was just visible at the apical foramen and then 0.5 mm was subtracted from the measured length. A standardized step-back technique using K-files was used. Each root canal was enlarged to #35 apical file with 2.25% NaOCl irrigation. After canal instrumentation, all specimens were dried with absorbent paper points and filled with gutta-percha (Aria Dent, Tehran, Iran) and root canal sealer (AH-Plus, Dentsply De-Tray, Konstanz, Germany) using cold lateral condensation technique. Gutta-percha was removed to 5 mm of the working length with a heat carrier.The crownswere then cut off with a diamond disk so that the remaining length was adjusted to 15 mm. All the roots were embedded in acrylic blocks. After resin setting, a groove was made to mark the buccal root location and the blocks were mounted on dental arch.

### Residual Dentin Thickness (RDT) Measurement

To evaluate the samples using CBCT, they were mounted in putty (Exaplast Set, Detax, Ettlingen, Germany). The exposure conditions of CBCT (Promax, Planmeca, Helsinki, Finland) set in image scanning were: MA=8; KVP=72; high resolution. The center of the buccal root canal from coronal aspect was determined as zero point using Romexis software (Planmeca, Helsinki, Finland). Dentin thickness of both roots was measured at 4, 6 and 8 mm apical to this point. Measurements were made at 4 points (buccal, palatal, mesial and distal). After post space preparation, the teeth were returned to the putty frame and RDT was re-measured in a same manner previously described.

### Post Space Preparation

After initial scanning, the samples were randomly divided into two groups, mounted in a dental arch and then fixed on a phantom head. Post spaces were prepared in the coronal 10-mm of root canals with #2 and #3 Gates-Glidden burs (Dentsply, Maillefer, Switzerland) in group 1 and #1 and #2 Peeso reamer drills (Dentsply, Maillefer, Switzerland) in group 2.

### Statistical Analysis

ANOVA tests with repeated measures were used to determine significant differences in RDT between the various procedures at each root level with different measurement directions. Statistical significance was set at *P*<0.05.

## Results

In some slices, the buccal and palatal roots were not completely divided; therefore, measurements were made for the buccal, mesial and distal aspects of the buccal roots, and for the palatal, mesial and distal aspects of the palatal roots.

Means and standard deviations for RDT on each root canal wall at sectioned levels before and after preparation of post space preparation is shown in Table 1. Some apical slices had to be discarded due to blurred CBCT images. In all slices, the RDT of the mesial aspect was thinner than the distal aspect. In the apical slices, the inner walls were thinner compared to the outer walls. 

**Table 1.** Means (SD) of residual dentin thickness (RDT) on each root canal wall and at 3 selected levels before and after post space preparation with Gates Glidden (group 1) and Peeso reamer (group 2)

Root	Level	Root wall	Group 1		Group 2	
			Before (mm)	After (mm)	Before (mm)	After (mm)
**Buccal**	**Coronal**	B	1.99 (0.286)	1.92 (0.282)	1.95 (0.247)	1.86 (0.260)
		M	1.62 (0.271)	1.60 (0.210)	1.61 (0.237)	1.51 (0.216)
		D	1.70 (0.194)	1.65 (0.181)	1.74 (0.308)	1.63 (0.312)
	**Middle**	B	1.49 (0.254)	1.45 (0.269)	1.49 (0.234)	1.38 (0.221)
		M	1.38 (0.178)	1.33 (0.201)	1.46 (0.201)	1.28 (0.181)
		D	1.47 (0.269)	1.40 (0.284)	1.56 (0.246)	1.39 (0.229)
	**Apical**	B	1.19 (0.285)	1.12 (0.281)	1.17 (0.106)	1.02 (0.184)
		P	0.95 (0.383)	0.83 (0.407)	1.11 (0.280)	0.83 (0.278)
		M	1.22 (0.229)	1.13 (0.258)	1.22 (0.114)	1.05 (0.199)
		D	1.37 (0.227)	1.21 (0.227)	1.32 (0.187)	1.14 (0.174)
**Palatal**	**Coronal**	P	1.92 (0.287)	1.87 (0.243)	1.82 (0.271)	1.75 (0.229)
		M	1.49 (0.368)	1.43 (0.327)	1.45 (0.322)	1.38 (0.272)
		D	1.62 (0.238)	1.58 (0.242)	1.52 (0.200)	1.42 (0.229)
	**Middle**	P	1.56 (0.232)	1.48 (0.245)	1.49 (0.243)	1.38 (0.257)
		M	1.33 (0.292)	1.22 (0.323)	1.22 (0.181)	1.08 (0.205)
		D	1.39 (0.234)	1.30 (0.221)	1.38 (0.196)	1.23 (0.211)
	**Apical**	B	1.20 (0.301)	1.04 (0.319)	1.25 (0.332)	0.83 (0.367)
		P	1.45 (0.307)	1.33 (0.261)	1.42 (0.320)	1.09 (0.331)
		M	1.27 (0.272)	1.09 (0.494)	1.14 (0.163)	0.92 (0.188)
		D	1.41 (0.277)	1.16 (0.246)	1.29 (0.244)	1.12 (0.188)

**Figure 1.**
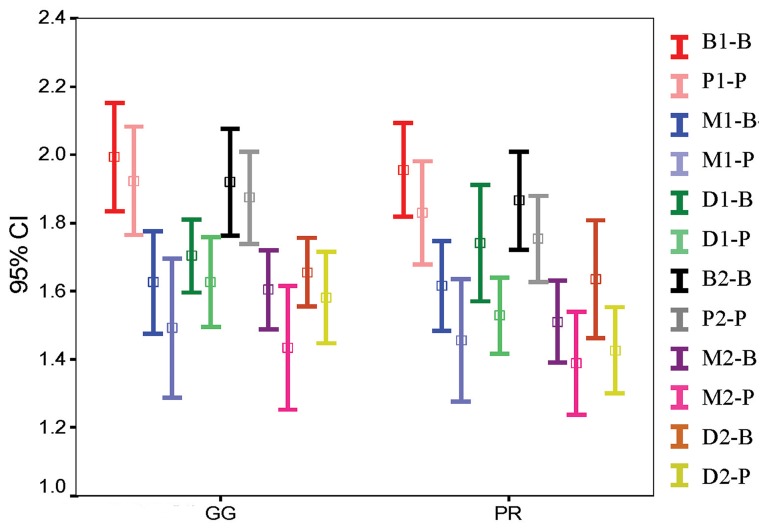
Mean RDT of root walls in relation to the aspect, stage, root and instrument type in coronal level from left to right: aspect code (B: Buccal, P: Palatal, M: Mesial and D: Distal), stage code (1: before and 2: after preparation) and root code (B: Buccal and P: Palatal root); GG: Gates Glidden; PR: Peeso Reamer

**Figure 2.**
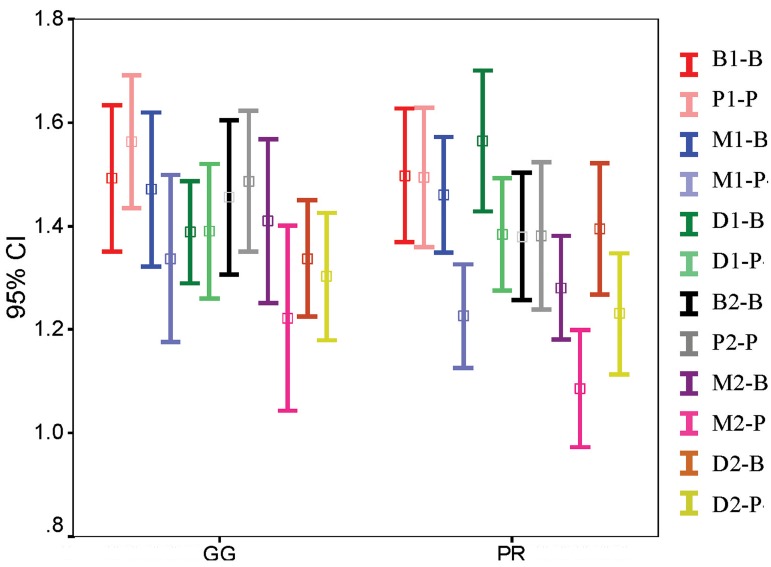
Mean RDT of root walls related to stage, aspect, root and instrument in middle level

**Figure 3.**
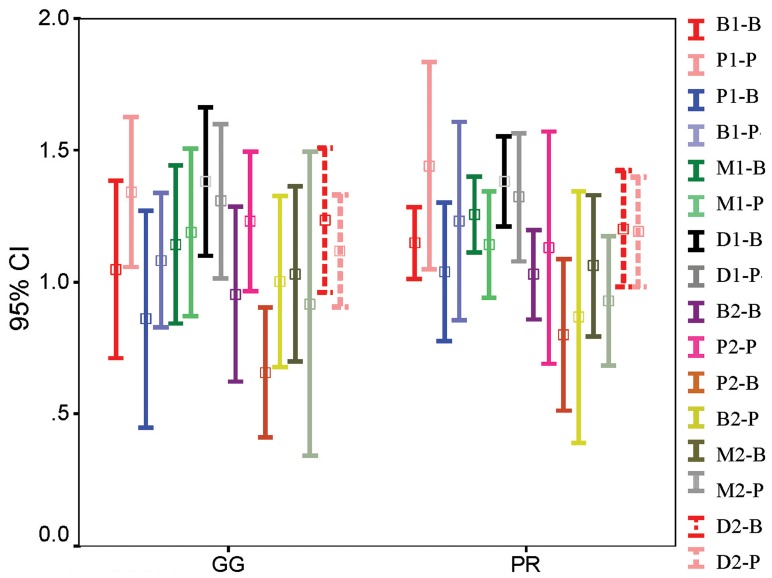
Mean RDT of root walls related to stage, aspect, root and instrument in apical level

Four-way ANOVA results with repeated measures for the independent variables stage (before or after post space preparation), aspect (M, B, D, P), root (B, P) and instrument (GG or P) are shown in Figures 1, 2 and 3. In all of the sections, differences were significant with regard to stage (*P*=000) and aspect (*P*<0.05). Differences in RDT were also significant for root variable in the coronal and middle sections (*P*<0.05). The interactions of stage × instrument in coronal sections, stage × instrument and aspect × root in the middle sections and aspect × root in the apical sections were also significantly different. The three and four-way interactions between variables were not statistically significant.

## Discussion

The residual dentin thickness after root canal and post space preparation is very important. Excessive removal of radicular dentin weakens the root [14] and might result in perforations and VRF [15-17]. The most common form of the maxillary first premolar is the two-rooted form [18], and in the most of two-rooted teeth, furcation area is located in the middle third of them [19], therefore, the present study was conducted on two-rooted maxillary first premolars with bifurcation in the middle third.

Most of the previous studies on RDT after post space preparation [13, 20-22] were carried out under ideal conditions; the samples were hand-held with optimal access to the canals and without interference by gutta-percha. In the present study, after obturating the canals with gutta-percha, the samples were mounted on a dental arch and fixed on phantom head to simulate clinical conditions.

Pilo *et al.* reported that post space preparations with parallel-sided drills such as ParaPost jeopardizes root integrity due to RDT values of less than 1 mm in most situations; therefore, Gates-Glidden and Peeso reamer drills were used for dowel preparation in this study [21].

It is recommended that 5 mm of gutta-percha should be retained apically [23]. In the present study, post spaces were prepared in the coronal 10 mm of root canals and 3 horizontal root post space levels were evaluated. In the apical sections of both buccal and palatal roots of one specimen and in the palatal roots of two specimens, gutta-percha had remained after dowel preparation. These slices had to be discarded because the remaining gutta-percha resulted in blurred images. Achieving a desired length for post space preparation in such cases was limited due to canal curvature (especially in the palatal roots) and relative inflexibility of GG and Peeso reamer drills.

Several techniques have been described to evaluate the root canal wall thickness. Souza *et al.* showed that radiographic measurements overestimate the root canal wall thickness by approximately 25% [20, 24-27]. Muffle technique [16, 20] has also some limitations such as invasive nature and destructive sectioning of the specimens and requirement of physical reassembly of sections. Cone beam CT has become popular for measurements of dentin thickness of root canal walls. Hartmann *et al.* showed that this technique can be reliable, without destructive sectioning of the specimens [25]. Ozgur Uyanik *et al.* demonstrated that CT scans allow easy measurement of canal changes and decrease the potential of radiographic or photographic transfer error [26].

In the present study, the RDT gradually decreased as the distance increased apically from the CEJ, which is consistent with previous studies [13, 21, 22].

After post space preparation, mean RDT of the palatal aspect of the buccal root in the apical sections was less than the recommended 1 mm (0.75 and 0.86 mm in groups 1 and 2, respectively). These values correspond to RDT values of 0.82- 0.9 mm reported by Pilo *et al.* [21] and 0.68 mm by Katz *et al.* [13]. The slight difference in mean valuesmight was attributed to the more coronal or apical location of the slice in these studies.

Minimal RDT values, before and after dowel preparation, were also recorded in the palatal walls of the buccal roots (0.45 and 0.36 mm, respectively), consistent with Pilo *et al’s. *results [21]. Mean reduction in RDT after dowel preparation was also greater in this aspect (0.2 and 0.25 mm in groups 1 and 2, respectively). In the apical sections in which the buccal and palatal roots were completely divided, the inner aspects of both roots were thinner compared to the outer aspects, consistent with the results of previous studies [13, 21]. Pilo *et al.* mentioned that the furcation groove in the buccal root and the divergence of the canal below the bifurcation point in the palatal root may be responsible for this significant interaction [21].The significant interaction of stage × instrument in the coronal and middle sections implied that decrease in RDT is depended on the instrument type. G.G with shorter blade and thinner shaft is more flexible compared to Peeso reamer. Furthermore, it is assumed that the shorter spiral cutting blades of GG might reduce dentinal contact and result in greater RDT after post space preparation.

## Conclusion

The clinician should be aware of specific natural anatomy in bifurcated maxillary first premolars because even a conservative approach in post space preparation might jeopardize root integrity. In buccal roots, dowel preparation should be avoided where possible. When post space preparation is mandatory, it seems that the use of Gates-Glidden drills is preferable to Peeso reamers.
